# Association between Epstein-Barr virus infection and gastric cancer: a systematic review and meta-analysis

**DOI:** 10.1186/s12885-020-07013-x

**Published:** 2020-06-01

**Authors:** Ahmad Tavakoli, Seyed Hamidreza Monavari, Farid Solaymani Mohammadi, Seyed Jalal Kiani, Saber Armat, Mohammad Farahmand

**Affiliations:** 1 Research Center of Pediatric Infectious Diseases, Institute of Immunology and Infectious Diseases, Iran University of Medical Sciences, Tehran, Iran; 2 Department of Medical Virology, Faculty of Medicine, Iran University of Medical Sciences, Tehran, Iran; 3 Department of Biological Sciences, North Dakota State University, Fargo, North Dakota USA; 4 Department of Biology, College of Science, Shiraz University, Shiraz, Iran; 5 Department of Virology, School of Public Health, Tehran University of Medical Sciences, Tehran, Iran

**Keywords:** Epstein-Barr virus, EBV, Gastric cancer, Stomach cancer, Gastric carcinoma, Meta-analysis

## Abstract

**Background:**

Numerous studies conducted over the past 30 years have pointed to the presence of Epstein–Barr virus (EBV) in gastric cancer samples. This study was aimed to provide a meta-analytic review of the prevalence of EBV in gastric cancer patients, and to clarify the relationship between EBV infection and gastric cancer.

**Methods:**

A literature search was performed electronically using online databases for English language publications until July 1, 2019. The pooled EBV prevalence and 95% confidence intervals (CIs) were estimated using a random-effects model. To determine the association between EBV and gastric cancer, pooled odds ratio (OR) and its 95% CI were computed for case-control studies. Two separate analyses were performed on data from case-control studies with matched and non-match pairs designs to calculate the pooled estimates of ORs.

**Results:**

The pooled prevalence of EBV in 20,361 gastric cancer patients was 8.77% (95% CI: 7.73–9.92%; I^2^ = 83.2%). There were 20 studies with matched pairs design, including tumor and tumor-adjacent normal tissue pairs from 4116 gastric cancer patients. The pooled ORs were 18.56 (95% CI: 15.68–21.97; I^2^ = 55.4%) for studies with matched pairs design and 3.31 (95% CI: 0.95–11.54; I^2^ = 55.0%) for studies with non-matched pairs design. The proportion of EBV-associated gastric cancer among male cases was significantly higher than among female cases (10.83%, vs. 5.72%) (*P* < 0.0001). However, the pooled OR estimate for EBV-associated gastric cancer was significantly higher among females (21.47; 95% CI: 15.55–29.63; I^2^ = 0%) than in males (14.07; 95% CI: 10.46–18.93; I^2^ = 49.0%) (*P* = 0.06). EBV was more prevalent in the cardia (12.47%) and the body (11.68%) compared to the antrum (6.29%) (*P* = 0.0002).

**Conclusions:**

EBV infection is associated with more than 18 times increase the risk of gastric cancer. Although the prevalence of EBV was higher in male patients than in female patients with gastric cancer, women are more likely than men to develop EBV-associated gastric cancer. Our findings showed that using tumor-adjacent normal tissues as the control group provides more robust and accurate results regarding the relationship between EBV infection and gastric cancer.

## Background

According to GLOBOCAN statistics in 2018, gastric cancer is the fifth most frequently diagnosed cancer and the third leading cause of cancer-related mortality in the world accounted for 8.2% of all cancer deaths. Over 1,000,000 new cases of gastric cancer diagnosed in 2018 around the world, with an estimated 783,000 deaths [1]. Gastric cancer arises from a combination of multiple environmental and genetic risk factors, and infectious agents are one of the critical environmental factors which contribute to an increased risk of developing several malignancies [2].

Epstein-Barr virus (EBV), as a member of the *Herpesviridae* family, is the first described human cancer virus and is responsible for approximately 1.8% of all human cancers, including Hodgkin lymphoma, Burkitt lymphoma, NK/T cell lymphoma, and nasopharyngeal carcinoma [3]. However, the role of EBV in the development of other malignancies is still under investigation. At the beginning of the 1990s, the association between EBV and gastric carcinomas was found. The first report was made by Burke et al. in a case of lymphoepithelial-like gastric carcinoma [4], and afterwards, the association was observed in gastric adenocarcinoma [5]. Subsequently, numerous studies demonstrated an essential role of EBV in gastric carcinogenesis.

To date, the mechanisms of EBV-associated gastric cancer are still not comprehensively clarified. Generally, virologic aspects, in conjunction with host genome abnormalities, co-potentiate the cancer progression. Regarding the virologic background, the EBV genome encodes oncoproteins, which target important cellular pathways. EBV-associated gastric cancer belongs to latency type I infection, in which only EBNA1, EBER, BamHI A rightward transcript (BART), and BART miRNAs are highly expressed, while the latent membrane protein 2A (LMP2A) can be detected in 40% of cases [6]. Evidence suggests that latent infection by EBV and the expression of the EBV latent genes lead to the host genome abnormalities like aberrant DNA methylation, which has attracted more attention in recent years [7].

The gold standard for the diagnosis of EBV infection in histopathologic samples is ISH, which detects EBV-encoded small RNA-1 (EBER1). EBER1 is highly expressed in latently EBV-infected cells (up to 10^7^ copies per cell) [8]. EBER1 signals are commonly identified in the nuclei of nearly all carcinoma cells in EBV-associated gastric carcinoma [9]. PCR-based methods are also widely used for the diagnosis of EBV infection. Although PCR is a cost-efficient and simple technique for the detection of EBV infection, it is prone to false-positive results due to its low specificity. The low specificity of PCR can be explained by the fact that memory cells and/or non-tumor, bystander lymphocytes may also be investigated for the presence of the EBV genome. Therefore, PCR-based methods are more sensitive but less specific than the gold standard ISH method to detect EBV [10, 11].

There are several published meta-analyses addressing the prevalence of EBV among gastric cancer patients [12–16], however, their results are out of date and only descriptive. On the other hand, they did not perform any analysis to estimate the association between the EBV and gastric cancer risk. The last meta-analysis conducted by Bae et al. focused on the results of case-control studies published up to 2014 to prove the relationship between EBV and gastric cancer for the first time [17]. However, some important variables such as gender, type of samples, and tumor anatomical location did not include in their meta-analysis. Our meta-analysis aims to determine the association of EBV infection with gastric cancer and to provide an updated pooled prevalence of EBV infection among gastric cancer patients. It is anticipated that the results of the present study will direct future experimental studies toward elucidating the role of EBV infection in the carcinogenesis of gastric cancer, and will inform clinicians and policy-makers to improve preventive intervention and control.

## Methods

The present systematic review and meta-analysis was performed according to the recommendations of the Preferred Reporting Items for Systematic Reviews and Meta-Analyses (PRISMA) statement [18].

### Search strategy

A rigorous literature search was conducted using PubMed, Web of Science, Scopus, EMBASE, and Google scholar to identify all published articles reporting the prevalence of EBV in patients with gastric cancer. Databases were searched from inception to July 1, 2019. The bibliographies of all articles obtained were also reviewed for additional relevant publications. The list of keywords used for this systematic review and meta-analysis is provided in Additional file 1.

### Study selection

All records were imported to EndNote software version X8 (Thomson Reuters, California, USA), and duplicate entries were removed. The screening of the title and abstract of the remaining records was independently conducted by two researchers. The full-texts of the remaining records were then retrieved and reviewed, and any disagreements were resolved through discussion by a third investigator.

### Eligibility criteria

Studies were considered eligible for inclusion in the present meta-analysis, if they met the following criteria: (1) Studies using cross-sectional and case-control designs reporting the prevalence of EBV infection in patients with different types of gastric carcinoma; (2) Studies using EBER-ISH technique to detect the presence of EBV transcripts or nucleic acids; (3) Studies using the formalin-fixed paraffin-embedded (FFPE) tissues and biopsies samples; (4) Studies published in peer-reviewed journals in the English language.

Studies with following characteristics were excluded from the present meta-analysis: (1) Studies using serological techniques such as enzyme-linked immunosorbent assay (ELISA) to detect circulating antibodies to EBV infection; (2) Studies evaluating the presence of EBV in serum, plasma or peripheral blood mononuclear cell (PBMC) samples; (3) Studies assessing the presence of EBV in gastric carcinoma patients with underlying disorders; (4) Studies evaluating the presence of EBV by molecular methods such as PCR, nested-PCR and Real-Time PCR; (5) Studies addressing remnant gastric cancer, gastric lymphoma, and other types of gastric malignancies; (6) Studies using techniques other than EBER-ISH, (7) Studies published in languages other than English; (8) Reviews, letters to the editor, abstracts, and case reports.

### Data extraction and quality assessment

Two investigators independently extracted data from all eligible studies in a pre-designed data extraction form using Microsoft Excel 2013 (Microsoft Corporation, Redmond, Washington, USA). The two investigators cross-checked each other’s data extraction, and any disagreements were resolved by a third investigator. After retrieving the eligible articles, a modified checklist based on the guidelines of the strengthening the reporting of observational studies in epidemiology (STROBE) was used for assessing the risk of bias of the included studies [19, 20]. The checklist includes 12 questions that cover different methodological aspects. According to the checklist, the highest score was 12, representing the highest quality, and the minimum acceptable score was 8. Lastly, studies obtained the minimum score, and more were considered eligible to include in the main meta-analysis. The following characters were extracted from each study: first author’s name, publication date, study location, study design, sample size, sex, type of specimen, histological type, number of EBV-positive samples, tumor anatomical location, depth of invasion, tumor stage, and lymph node invasion.

### Statistical analysis

The present meta-analysis had two primary purposes; first, providing an updated estimate of the pooled prevalence of EBV among patients with gastric cancer, and secondly, investigating the association between EBV and the development of gastric cancer. A random-effect meta-analysis using the inverse variance method was applied to estimate the pooled prevalence of EBV (DerSimonian-Laird method) [21]. The logit transformation was used for stabilizing the variance and data normalization, and the Clopper-Pearson method was applied to determine the 95% confidence intervals (CIs) for proportions [22].

To evaluate the strength of the association between EBV infection and gastric cancer risk, the pooled odds ratios (ORs) with 95% CIs were generated from a random-effects model based on the DerSimonian-Laird method. For studies with a zero cell, a continuity correction of 0.5 was applied. We also conducted subgroup analyses to identify the possible sources of heterogeneity. The heterogeneity among the studies was assessed through I^2^ statistics [23]. To explore potential publication bias and symmetric assumption among the included studies, a Begg’s funnel plot was constructed [24]. All the above-mentioned analyses were conducted using the R package “meta” (version 3.5.3 [2019-03-11]) [25, 26], and *P* values less than 0.05 were considered statistically significant. Furthermore, for each case-control study with matched pairs design, we separately computed matched-pairs OR and its corresponding variance using the “escalc” function in the R “metafor” package [27] (version 2.1–0 [2019-05-13]. The obtained results were then used for performing meta-analysis to calculate the matched pairs pooled OR.

## Results

### Literature selection

**Fig. 1:**
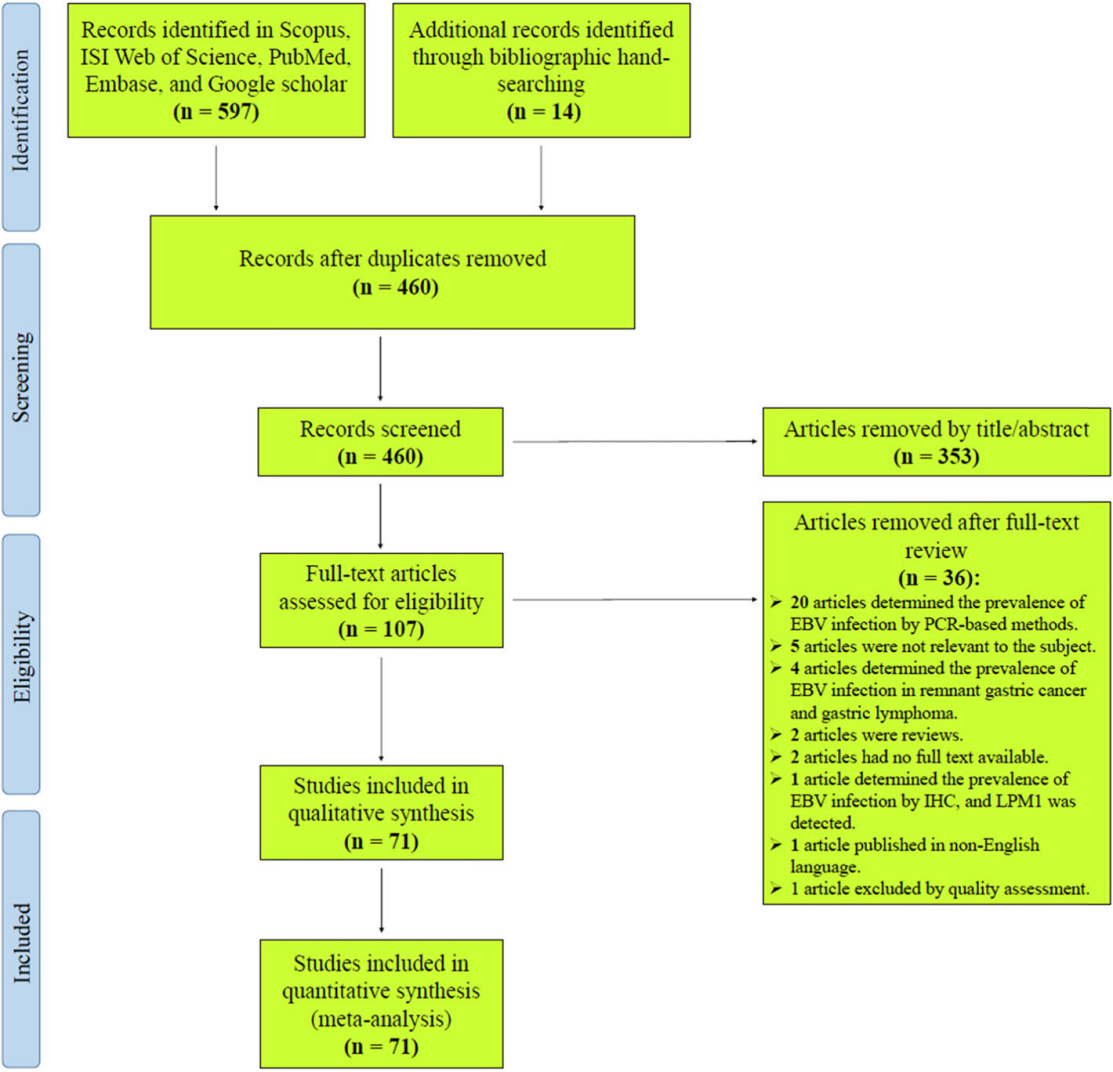
Flowchart presenting the steps of literature search and selection

The electronic database searches were identified 597 articles, and additional 14 relevant records were found through bibliographic hand searching. Of these 611 articles, 151 duplicates were excluded, so a total of 460 articles was screened according to their title and abstract. A total of 353 articles was eliminated after reading the title and abstract due to apparent irrelevance. The remaining 107 articles were assessed for agreement with the inclusion and exclusion criteria by the full-text review, and 72 papers met the scope criteria. Based on the modified STROBE checklist, 71 papers were deemed to have good quality (obtained scores of 8 and above), and only one paper [28] was failed to reach score 8. Finally, 71 papers were included in this systematic review and meta-analysis. Figure 1 shows the process of literature retrieval and screening using a flow chart.

### Study characteristics

Table 1 shows the characteristics of eligible studies included in the systematic review and meta-analysis. Out of 71 studies, 30 were case-control, and 41 were cross-sectional in design. Publication dates ranged from 1993 to 2019, and over half of the studies (59.1%) described specimens recruited before 2005. Among the studies included in this meta-analysis, four were from Africa, 16 were from America, 35 were from Asia, and 17 were from Europe. Of the 72 studies included, 46 provided information on patients’ sex, 40 studies provided data on histological type, and 35 had data on tumor anatomical location. The most extensive study included 2226 gastric cancer cases, and the smallest covered 19 cases. Most studies were from Japan (*n* = 15).

**Table 1:** Characteristics of the included studies in this systematic review and meta-analysis

Author [Ref.]	Year	Location	Study design	Type of sample	No. of case	No. of control	No. of case positive	No. of control positive
Rowlands [29]	1993	UK and Japan	Cross-sectional	FFPE	174		9	
Shibata [30]	1993	USA	Cross-sectional	FFPE	187		19	
Tokunaga [31]	1993	Japan	Cross-sectional	FFPE	1848		122	
Tokunaga [32]	1993	Japan	Cross-sectional	FFPE	999		69	
Imai [33]	1994	Japan	Case-control	FFPE	1000	1000	70	0
Ott [34]	1994	Germany	Case-control	FFPE	39	39	7	0
Shousha [35]	1994	UK	Case-control	FFPE	19	9	1	5
Yuen [36]	1994	China	Case-control	FFPE	74	36	7	0
Harn [37]	1995	Taiwan	Case-control	FFPE	55	49	6	0
Gulley [38]	1996	USA	Case-control	FFPE	95	95	11	0
Moritani [39]	1996	Japan	Case-control	FFPE	132	132	15	0
Selves [40]	1996	France	Case-control	FFPE	59	59	5	0
Shin [41]	1996	South Korea	Case-control	FFPE	89	37	12	0
Galetsky [42]	1997	Russia	Case-control	FFPE	206	206	18	0
Clark [43]	1997	Singapore	Cross-sectional	FFPE	137		6	
Ojima [44]	1997	Japan	Cross-sectional	FFPE	412		83	
Yanai [45]	1997	Japan	Cross-sectional	FFPE	124		12	
Herrera-Goepfert [46]	1999	Mexico	Cross-sectional	FFPE	135		11	
Kume [47]	1999	Japan	Case-control	FFPE	344	344	40	0
Takano [48]	1999	Japan	Cross-sectional	FFPE	513		33	
Wan [49]	1999	China	Case-control	FFPE	58	58	6	0
Chapel [50]	2000	France	Case-control	FFPE	56	56	7	0
Wu [51]	2000	Taiwan	Cross-sectional	Biopsy	150		30	
Corvalan [52]	2001	Chile	Case-control	FFPE	185	185	31	0
Kijima [53]	2001	Japan	Cross-sectional	FFPE	313		23	
Ishii [54]	2001	Japan	Cross-sectional	FFPE	119		23	
Koriyama [55]	2001	Brazil	Cross-sectional	FFPE	300		24	
Luqmani [56]	2001	UK	Case-control	FFPE	20	79	1	9
Burgess [57]	2002	UK	Cross-sectional	FFPE	534		9	
Kang [58]	2002	South Korea	Cross-sectional	FFPE	233		21	
Kattoor [59]	2002	India and Japan	Cross-sectional	FFPE	2226		135	
Vo [60]	2002	USA	Cross-sectional	FFPE	107		11	
Czopek [61]	2003	Poland	Cross-sectional	FFPE	40		5	
Oda [62]	2003	Japan	Case-control	FFPE	97	97	5	0
Ishii [63]	2004	Japan	Case-control	FFPE	133	133	19	0
Lee [64]	2004	South Korea	Cross-sectional	FFPE	1127		63	
Lopes [65]	2004	Brazil	Case-control	FFPE	53	53	6	0
van Beek [66]	2004	Netherlands	Cross-sectional	FFPE	566		41	
Alipov [67]	2005	Kazakhstan	Case-control	FFPE	139	139	14	0
Herrera-Goepfert [68]	2005	Mexico	Case-control	FFPE	330	330	24	2
Luo [69]	2005	China	Case-control	FFPE	172	172	11	0
Yoshiwara [70]	2005	Peru	Cross-sectional	FFPE	254		10	
Campos [71]	2006	Colombia	Cross-sectional	FFPE	368		42	
Szkaradkiewicz [72]	2006	Poland	Cross-sectional	FFPE	32		14	
Luo [73]	2006	China	Cross-sectional	FFPE	185		13	
von Rahden [74]	2006	Germany	Case-control	FFPE	82	82	5	0
Abdirad [75]	2007	Iran	Cross-sectional	FFPE	273		9	
Jung [76]	2007	South Korea	Cross-sectional	FFPE	111		7	
Lima [77]	2008	Brazil	Cross-sectional	FFPE	71		6	
Ryan [78]	2009	USA	Cross-sectional	FFPE	113		11	
Trimeche [79]	2009	Tunisia	Cross-sectional	FFPE	96		4	
Truong [80]	2009	USA	Case-control	FFPE	235	72	12	0
Ferrasi [81]	2010	Brazil	Case-control	FFPE	54	54	5	0
Koriyama [82]	2010	Japan	Cross-sectional	FFPE	156		21	
Chen [83]	2010	China	Case-control	FFPE	676	676	45	3
Boysen [84]	2011	Denmark	Cross-sectional	FFPE	131		10	
BenAyed-Guerfali [2]	2011	Tunisia	Cross-sectional	FFPE	81		12	
de Lima [85]	2012	Brazil	Cross-sectional	FFPE	160		11	
Ksiaa [86]	2014	Tunisia	Cross-sectional	FFPE	43		4	
Aslane [87]	2016	Algeria	Case-control	FFPE	97	10	22	0
Tsai [88]	2016	Taiwan	Cross-sectional	FFPE	1039		52	
Zhang [89]	2016	China	Cross-sectional	FFPE	600		30	
Liu [90]	2016	China	Case-control	FFPE	206	206	15	0
Na [91]	2017	South Korea	Cross-sectional	FFPE	205		15	
Boger [92]	2017	Germany	Cross-sectional	FFPE	484		22	
Kim [93]	2017	South Korea	Case-control	FFPE	207	56	13	0
Nogueira [94]	2017	Portugal	Case-control	FFPE	82	33	9	1
Ribeiro [3]	2017	Portugal	Cross-sectional	FFPE	179		15	
de Souza [95]	2018	Brazil	Cross-sectional	Biopsy	302		62	
Wanvimonsuk [96]	2018	Thailand	Case-control	FFPE	33	55	4	0
Martinez-Ciarpaglini [97]	2019	Spain	Cross-sectional	FFPE	209		13	

*FFPE* Formalin-Fixed Paraffin-Embedded

### The prevalence of EBV among gastric cancer patients

**Fig. 2:**
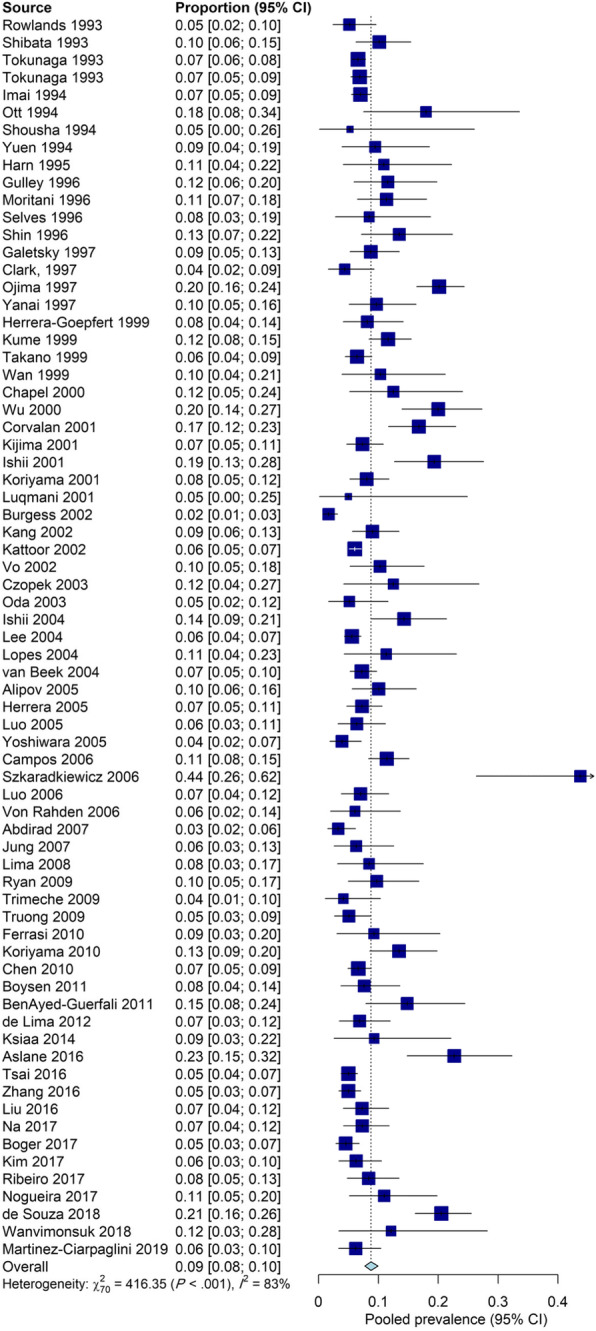
Forest plot of the prevalence of EBV infection among gastric cancer patients, according to the random effect model

**Fig. 3:**
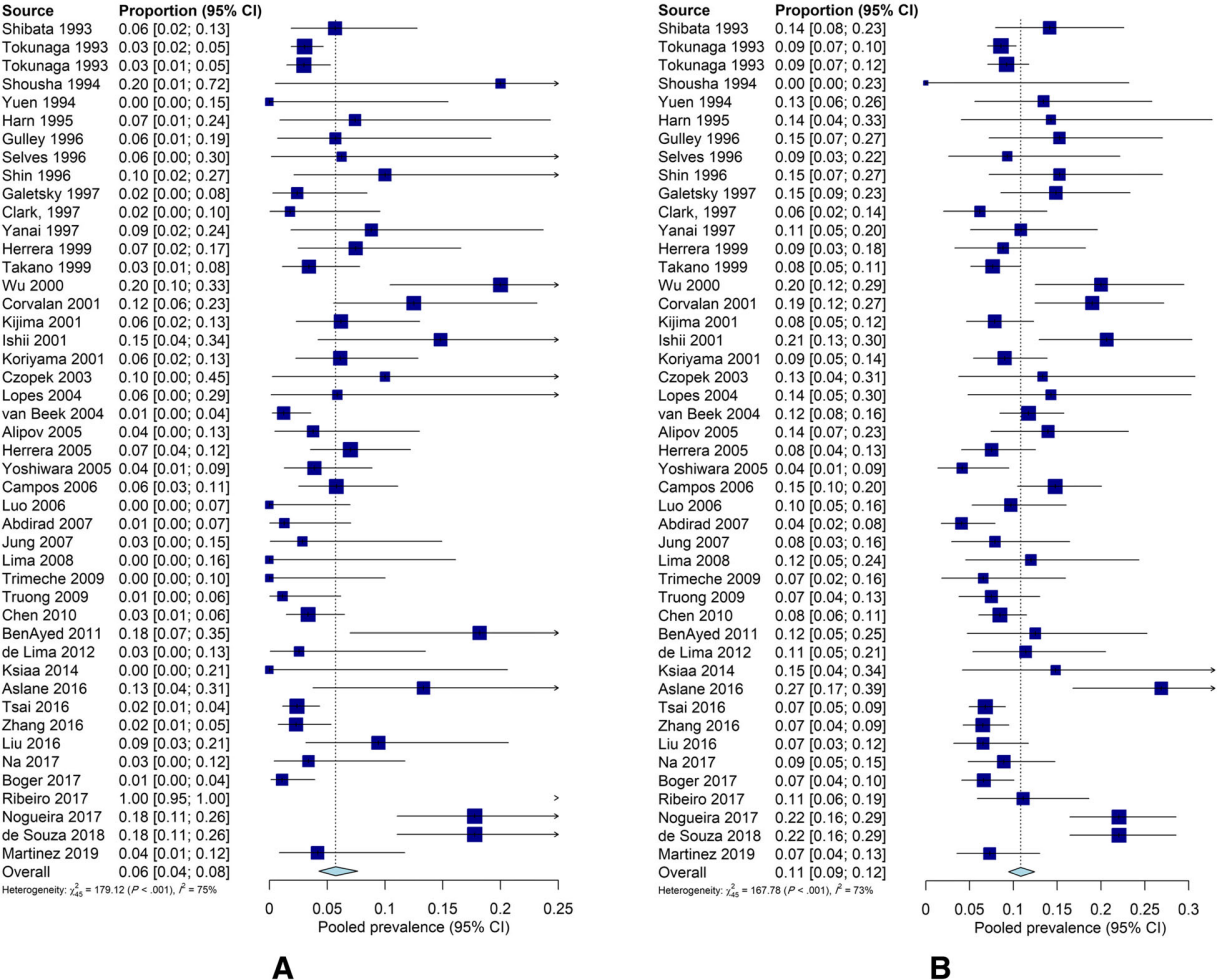
Forest plot of the prevalence of EBV infection among gastric cancer patients, according to the random effect model in females (**a**) and males (**b**)

The first aim of the current study was to determine the pooled prevalence of EBV in 20,361 gastric cancer patients from 26 countries, and the range was from 1.69 to 43.75% of the selected individual studies. Figure 2 shows the prevalence of EBV and 95% CI estimates from individual studies according to the random-effects model. The pooled prevalence of EBV among gastric cancer patients was 8.77% (95% CI: 7.73–9.92%; I^2^ = 83.2%). The highest and lowest prevalence of EBV were found in gastric cancer patients from Poland and the United Kingdom, respectively (25.57, 95%CI: 6.13–64.36% vs. 2.78, 95%CI: 1.51–5.06%). The proportion of EBV-positive gastric cancer among male cases was significantly higher than among female cases (10.83, 95%CI: 9.43–12.40% vs 5.72, 95%CI: 4.27–7.64%) (*P* < 0.0001) (Fig. 3). Table 2 presents more detailed information on the prevalence of EBV infection in gastric cancer patients for subgroups.

**Table 2:** Subgroup analysis of the prevalence of EBV infection in gastric cancer patients

Characteristics	Categories	No. of Studies	Pooled prevalence (%) (95% CI)	Heterogeneity test I^**2**^%, ***p***-value	Differences between subgroups; χ^**2**^ test (***p***-value)
**Overall**	–	71	8.77 (7.73–9.92)	83.2%, *P* < 0.01	–
**Study design**	Cross-sectional	41	8.22 (6.90–9.77)	88.2%, *P* < 0.01	*P =* 0.15
	Case-control	30	9.71 (8.32–11.30)	59.4%, *P* < 0.01	
**Publication date**	≤2005	42	8.91 (7.65–10.35)	82.5%, *P* < 0.01	*P =* 0.77
	> 2005	29	8.56 (6.81–10.71)	84.6%, *P* < 0.01	
**Sex**	Male	46	10.83 (9.43–12.40)	73.2%, *P* < 0.01	*P* < 0.0001†
	Female	46	5.72 (4.27–7.64)	74.9%, *P* < 0.01	
**Study location**	Africa	4	11.93 (5.97–22.44)	76.8%, *P* < 0.01	*P =* 0.64
	America	16	9.51 (7.45–12.07)	76.8%, *P* < 0.01	
	Asia	35	8.38 (7.15–9.80)	84.1%, *P* < 0.01	
	Europe	17	8.21 (5.82–11.46)	80.4%, *P* < 0.01	
**Development status**	Developed countries	34	8.42 (7.11–9.94)	82.1%, *P* < 0.01	*P =* 0.64
	Developing countries	39	8.92 (7.40–10.73)	83.5%, *P* < 0.01	
**Sample type**	FFPE	69	8.49 (7.54–9.55)	79.9%, *P* < 0.01	*P* < 0.0001†
	Biopsy	2	20.36 (16.89–24.32)	0%, *P* = 0.9	
**Lauren’s histological type**	Intestinal type	40	8.10 (6.64–9.83)	69.2%, *P* < 0.01	*P =* 0.31
	Diffuse type	40	9.41 (7.54–11.69)	77.0%, *P* < 0.01	
**Tumor anatomical location**	Cardia	32	12.47 (10.39–14.89)	24.8%, *P* = 0.1	*P =* 0.0002†
	Body	32	11.68 (9.96–13.65)	32.0%, *P* = 0.04	
	Antrum	35	6.29 (4.67–8.42)	76.8%, *P* < 0.01	
**Depth of invasion**	Early	7	13.00 (9.20–18.06)	0%, *P = 0.71*	*P* = 0.45
	Advanced	7	10.80 (7.64–15.06)	58.1%, *P* = 0.03	
**Tumor stage**	I + II	14	7.39 (5.79–9.39)	29.5%, *P* = 0.14	*P* = 0.36
	III + IV	14	8.80 (6.57–11.68)	64.4%, *P* < 0.01	
**Lymph node invasion**	Absent	14	8.75 (6.02–12.55)	57.9%, *P* < 0.01	*P* = 0.91
	Present	14	9.00 (6.33–12.65)	77.4%, *P* < 0.01	

*FFPE* Formalin-Fixed Paraffin-Embedded

†Statistically significant

### The association between EBV and gastric cancer

**Fig. 4:**
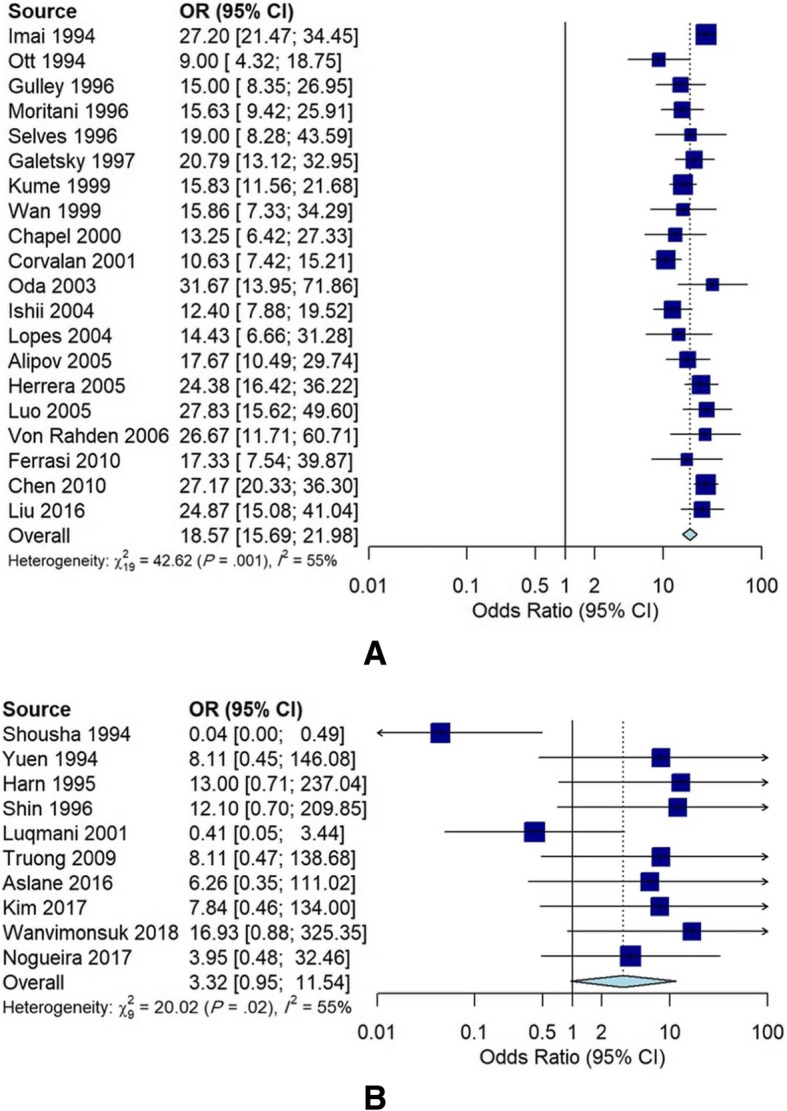
Forest plot of the association between EBV infection and gastric cancer risk (according to random effect model) in studies with match pairs design (**a**) and non-match pairs design (**b**)

**Fig. 5:**
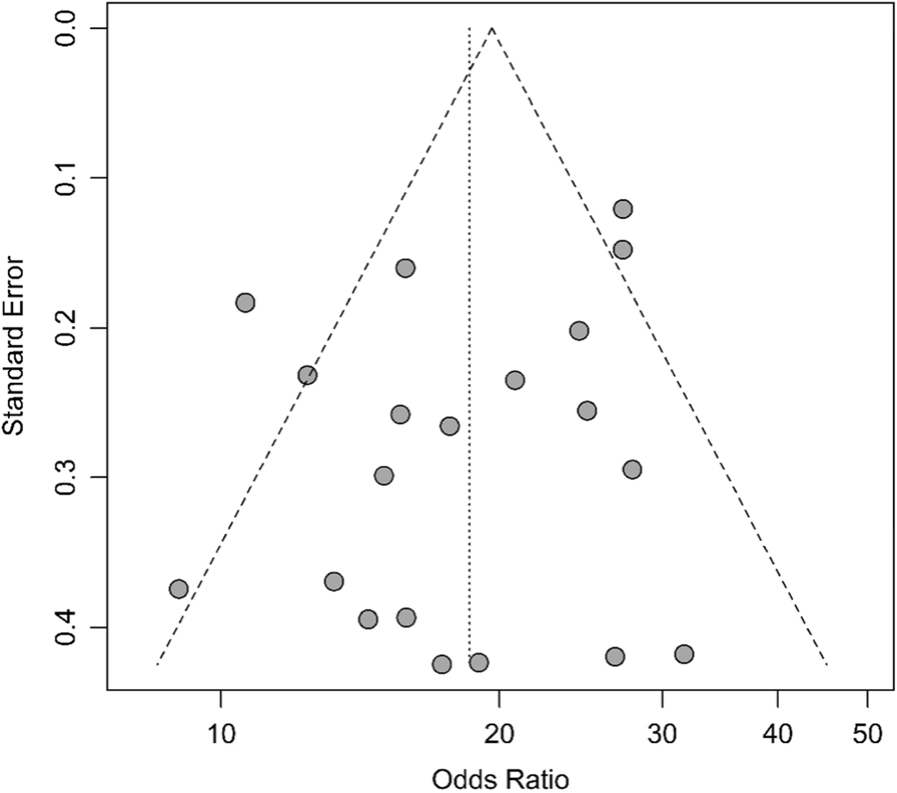
Funnel plot for assessment of publication bias

Among 30 case-control studies, 20 had matched pairs design, including tumor and tumor-adjacent normal tissue pairs from 4116 gastric cancer patients. The remaining ten non-matched case-control studies included 911 cases of gastric cancer and 436 controls. Using data obtained from studies with non-matched pairs design, the pooled OR of EBV infection was 3.31 (95% CI: 0.95–11.54; I^2^ = 55.0%), whereas the pooled OR for studies with matched pairs design was 18.56 (95% CI: 15.68–21.97; I^2^ = 55.4%), indicating a solid significant positive relationship between EBV infection and gastric cancer (Fig. 4). So, we further performed a subgroup analysis for studies with matched pairs design. Table 3 presents details on the association between EBV infection and gastric cancer risk for subgroups. Finally, the analysis of the funnel plot did not show evidence of asymmetry (Fig. 5), and Begg’s test indicated an absence of publication bias among all the studies included in this meta-analysis (*P* = 0.18).

**Table 3:** Subgroup analysis of association between EBV infection and gastric cancer risk

Characteristics	Categories	No. of Studies	Pooled OR (95% CI)	Heterogeneity test I^**2**^%, ***p***-value	Differences between subgroups; χ^**2**^ test (***p***-value)
**Overall**	–	20	18.56 (15.68–21.97)	55.4%, *P* < 0.01	–
**Sex**	Male	8	14.07 (10.46–18.93)	49.0%, *P* = 0.06	*P* = 0.06
	Female	8	21.47 (15.55–29.63)	0%, *P* = 0.55	
**Study location**	America	5	15.69 (10.82–22.74)	57.4%, *P* = 0.05	*P* = 0.33
	Asia	9	21.00 (16.77–26.30)	59.1%, *P* = 0.01	
	Europe	6	17.23 (13.19–22.51)	5.6%, *P* = 0.38	
**Development status**	Developed countries	10	17.31 (13.38–22.40)	58.7%, *P* < 0.01	*P* = 0.46
	Developing countries	10	19.73 (15.56–25.03)	56.2%, *P* = 0.01	
**Lauren’s histological type**	Intestinal type	10	15.07 (9.55–23.78)	62.0%, *P* < 0.01	*P* = 0.27
	Diffuse type	10	10.69 (7.14–16.00)	79.0%, *P* < 0.01	
**Tumor anatomical location**	Cardia	10	6.65 (5.18–8.52)	21.8%, *P* = 0.24	*P* = 0.46
	Body	10	6.31 (2.38–16.69)	97.0%, *P* < 0.01	
	Antrum	11	15.55 (4.12–58.62)	98.2%, *P* < 0.01	
**Depth of invasion**	Early	3	5.87 (2.78–12.40)	45.8%, *P = 0.16*	*P* < 0.01†
	Advanced	3	19.94 (13.31–29.85)	22.9%, *P* = 0.27	
**Tumor stage**	I + II	2	33.50 (10.85–103.46)	73.8%, *P* = 0.05	*P* = 0.52
	III + IV	2	22.26 (13.05–37.96)	24.6%, *P* = 0.25	
**Lymph node invasion**	Absent	3	16.98 (9.02–31.95)	1.3%, *P* = 0.36	*P* = 0.58
	Present	3	23.21 (9.44–57.03)	80.6%, *P* < 0.01	

† Statistically significant

## Discussion

Our meta-analysis showed that the pooled prevalence of EBV among gastric cancer patients from 26 countries is 8.77% (95% CI: 7.73–9.92%; I^2^ = 83.2%). We chose strict inclusion and exclusion criteria to obtain pertinent studies and to increase the chance of finding a valid conclusion. The pooled prevalence and OR obtained in this meta-analysis were calculated from studies that detected EBV infection with the ISH method. All studies that investigated the presence of EBV by other methods, including different types of PCR assays, and even immunohistochemistry (IHC), did not consider in our analysis. The reason for this stems from the fact that the sensitivity and specificity of each detection method are different, and it is not reliable to draw a conclusion using the pooled data.

The gold standard technique for the detection of EBV in tissues is ISH with EBV EBERs (EBER-ISH) due to its high sensitivity and specificity to determine the precise intranuclear localization of the EBV-infected cells. The diagnosis of EBV-associated gastric cancer is confirmed by the presence of EBER within the tumor cells and its absence in the normal tissue adjacent to the tumor [3]. Many studies have reported the higher prevalence of EBV among gastric cancer patients by PCR assay than the EBER-ISH technique [17]. However, PCR is unable to discriminate between cancer cells and lymphocytes infiltrating in tumor stromal, and thus it is impossible to know from where the EBV genome is amplified. It should be noted that the vast majority of people (nearly 90%) are EBV carriers, and their lymphocytes probably contain EBV genomes [11]. Regarding the statements above, our meta-analysis exclusively focused on the positivity of the EBV-associated gastric cancers by ISH only.

One of the major strong points in this meta-analysis is that the pooled estimates of ORs were calculated from studies with matched pairs and non-matched pairs designs, separately, with different statistical methods. The detailed descriptions about the analysis of data for matched pairs and non-matched pairs studies are available in several previous studies [98]. It has been recommended that a matched-pairs analysis should be used to assess effect sizes for studies with matched pairs design. Accordingly, the pooled OR determined for studies with non-matched pairs and matched pairs designs were 3.31 (95% CI: 0.95–11.54; I^2^ = 55.0%) and 18.56 (95% CI: 15.68–21.97; I^2^ = 55.4%), respectively. We performed two separate analyses for studies with match pairs and non-match pairs designs to demonstrate that the strength of association (ORs) between EBV infection and gastric cancer is the highest when tumor-adjacent normal tissues are used as a control group. This is due to the fact that confounding variables are eliminated from data analysis. Therefore, we can obtain more accurate and robust estimates of the association between EBV and gastric cancer. This finding of our study will be beneficial for researchers to design their future case-control studies appropriately. Using the tumor-adjacent normal tissues as the control group will provide more accurate results regarding the relationship between EBV infection and gastric cancer.

To date, several studies have attempted to discover the role of EBV infection in gastric cancer progression. EBV enters B lymphocytes in oropharyngeal lymphoid tissues. The virus then enters the gastric epithelial cells, either by the cell-to-cell contact between B lymphocytes and gastric epithelial cells or by direct entry into the gastric epithelia [99]. It has been reported that EBV entry into the gastric epithelial cells is facilitated by the previous mucosal damage [68]. After the virus enters the cell, EBV establishes type I latency in which a limited set of the latent gene is expressed [79]. A recent systematic review study showed that the most of the EBV latent proteins expressed in gastric cancer cases were EBNA1 (98.1%) and LMP2A (53.8%), whereas LMP1 and LMP2B were detected in only 10% of EBV-associated gastric cancer cases. Some of the lytic proteins, such as BARF1, were also reported to be present in almost half of EBV-associated gastric cancer cases [100]. It is shown that the EBV-encoded BARF1 acts as an oncogene and promotes cell proliferation in gastric cancer through upregulation of NF-κB signaling and reduction of the cell cycle inhibitor p21 [101]. It is well known that DNA methylation plays a crucial role in gastric cancer development and progression [102]. Methylation of both viral and cellular genome is one of the critical mechanisms involved in the development and maintenance of EBV-associated gastric cancer. It is well documented that EBV latent membrane protein 2A (LMP2A) plays a variety of key roles in the epigenetic abnormalities such as aberrant DNA methylation in host stomach cells, and the development and maintenance of EBV-associated gastric cancer [9].

Another interesting finding of our meta-analysis is that the prevalence of EBV was 1.9-fold higher in male patients than in female patients with gastric cancer (*P* < 0.0001). However, the OR estimate for EBV-associated gastric cancer was significantly higher among females than in males (*P* = 0.06). According to these results, we concluded that women are more likely than men (1.5-fold) to develop EBV-associated gastric cancer. This novel finding can be explained by different genetic backgrounds, lifestyles, or hormonal conditions between the two genders.

Subgroup analyses based on the tumor anatomical location indicate an anatomic preference for EBV during gastric carcinogenesis. Indeed, EBV-associated gastric cancers were significantly more prevalent in the cardia and the body of the stomach than in the antrum (*P* = 0.0002) (Table 2). However, the situation was different when OR was calculated. So that the OR estimate for EBV-associated gastric cancer was remarkably higher in the antrum than in the cardia and in the body (Table 3), although the difference was not statistically significant. This feature can be justified by the fact that the various parts of the stomach have different physiological conditions.

One prominent finding of the present meta-analysis is that EBV was detected more frequently in biopsy samples than in FFPE specimens from gastric cancer patients (2.4-fold, *P* < 0.0001). It is well documented that there are several challenges when working with FFPE samples, such as the low amount of extracted nucleic acids, and fragmentation of genomes and transcripts during the processes of fixation and embedding in paraffin. Therefore, to prevent false-negative results, using biopsy samples is recommended.

According to Lauren’s histological classification, gastric carcinoma is classified into two distinct types, namely intestinal and diffuse types. There are many differences between intestinal and diffuse types based on their epidemiology, etiology, and pathology [82]. However, the current meta-analysis showed that the prevalence of EBV was similar in intestinal and diffuse types (8.10 and 9.41%, respectively), and no significant association of EBV infection with the histological type was found (*P* = 0.31).

Similarly, our results did not indicate any significant difference in the prevalence of EBV-associated gastric cancer among different geographic regions, even between developed and developing countries. The same prevalence in developed and developing countries demonstrates that economic conditions are not related to EBV-associated gastric cancer risk.

There are some limitations in this study arose from the nature of the data sources used in the meta-analysis. Gastric cancer is a multifactorial disease affected by several risk factors. Age is considered as a risk factor for the development of EBV-associated gastric carcinoma. However, the majority of studies included in the current meta-analysis did not categorize EBV-infected and -uninfected gastric cancer patients based on the age group. Subsequently, we were not able to perform a subgroup analysis in this regard. Besides, there are some reports on the association between *Helicobacter pylori* infection and gastric cancer. Nevertheless, we did not consider data regarding the co-infection of EBV and *Helicobacter pylori*.

## Conclusions

To sum up, our meta-analysis suggests that the pooled prevalence of EBV among patients with gastric cancer was 8.77%. To determine the association between EBV infection and gastric cancer, a matched-pairs analysis from case-control studies was performed, and the pooled OR was calculated 18.56. This finding indicates a robust positive association between EBV infection and gastric cancer risk. We recommend using biopsy instead of FFPE samples and the ISH technique instead of PCR methods to ensure the validity of results.

Furthermore, the pooled prevalence of EBV was obtained from data from 26 countries in the world. Therefore, conducting studies in other geographical regions is strongly recommended to get more reliable estimates. Furthermore, we suggest that researchers use the tumor-adjacent normal tissues as the control group for their case-control studies to achieve more accurate results regarding the relationship between EBV infection and gastric cancer.

## Supplementary information

**Additional file 1.**

## Data Availability

All data generated or analyzed during this study are included in this article.

## Abbreviations

EBV: Epstein–Barr virus
CI: Confidence interval
OR: Odds ratio
EBER: EBV-encoded small RNA
ISH: In situ hybridization
PCR: Polymerase chain reaction
FFPE: Formalin-fixed paraffin-embedded
ELISA: Enzyme-linked immunosorbent assay
PBMC: Peripheral blood mononuclear cell
IHC: Immunohistochemistry
LMP: Latent membrane protein
BARF-1: BamH1-A Reading Frame-1
NF-κB: Nuclear factor kappa B
EBNA-1: Epstein–Barr nuclear antigen 1
