# Supplementary material for: Association between Epstein-Barr virus infection and gastric cancer: a systematic review and meta-analysis
Source: BMC Cancer. 2020 Jun 1;20:493. doi: 10.1186/s12885-020-07013-x (PMC7268387; doi:10.1186/s12885-020-07013-x)
Supplement: Supplementary file 1 — Additional file 1. [file 12885_2020_7013_MOESM1_ESM.docx]

**Appendix 1**

**Embase**

('cancer, stomach'/exp OR 'cancer, stomach' OR 'gastric cancer'/exp OR 'gastric cancer' OR 'stomach cancer'/exp OR 'stomach cancer') AND ('eb virus'/exp OR 'eb virus' OR 'epstein barr virus'/exp OR 'epstein barr virus' OR 'infectious mononucleosis herpetovirus'/exp OR 'infectious mononucleosis herpetovirus' OR 'barr epstein virus'/exp OR 'barr epstein virus' OR 'ebv'/exp OR 'ebv' OR 'epstein barr herpetovirus'/exp OR 'epstein barr herpetovirus' OR 'epstein virus'/exp OR 'epstein virus' OR 'epstein-barr virus'/exp OR 'epstein-barr virus' OR 'herpesvirus 4, human'/exp OR 'herpesvirus 4, human' OR 'human herpes virus 4'/exp OR 'human herpes virus 4' OR 'human herpesvirus 4'/exp OR 'human herpesvirus 4' OR 'infectious mononucleosis virus'/exp OR 'infectious mononucleosis virus' OR 'mononucleosis infectiosa virus'/exp OR 'mononucleosis infectiosa virus' OR 'virus, epstein barr'/exp OR 'virus, epstein barr' OR 'virus, infectious mononucleosis'/exp OR 'virus, infectious mononucleosis') AND ('case control study'/exp OR 'case control study' OR 'case-control studies'/exp OR 'case-control studies' OR 'case-control study'/exp OR 'case-control study' OR 'control study, case'/exp OR 'control study, case' OR 'matched case control'/exp OR 'matched case control' OR 'matched case control studies'/exp OR 'matched case control studies' OR 'matched case control study'/exp OR 'matched case control study' OR 'cross-sectional design'/exp OR 'cross-sectional design' OR 'cross-sectional research'/exp OR 'cross-sectional research' OR 'cross-sectional studies'/exp OR 'cross-sectional studies' OR 'cross-sectional study'/exp OR 'cross-sectional study' OR 'prevalence'/exp OR 'prevalence' OR 'prevalence study'/exp OR 'prevalence study' OR 'clinical epidemiology'/exp OR 'clinical epidemiology' OR 'epidemiologic factors'/exp OR 'epidemiologic factors' OR 'epidemiologic methods'/exp OR 'epidemiologic methods' OR 'epidemiologic research'/exp OR 'epidemiologic research' OR 'epidemiologic research design'/exp OR 'epidemiologic research design' OR 'epidemiologic studies'/exp OR 'epidemiologic studies' OR 'epidemiologic study characteristics'/exp OR 'epidemiologic study characteristics' OR 'epidemiologic study characteristics as topic'/exp OR 'epidemiologic study characteristics as topic' OR 'epidemiologic survey'/exp OR 'epidemiologic survey' OR 'epidemiological research'/exp OR 'epidemiological research' OR 'epidemiology'/exp OR 'epidemiology' OR 'detection, virus'/exp OR 'detection, virus' OR 'viral detection'/exp OR 'viral detection' OR 'virus detection'/exp OR 'virus detection') AND 'article'/it

**PubMed**

("Herpesvirus 4, Human"[Mesh] OR Burkitt Lymphoma Virus OR Lymphoma Virus, Burkitt OR Burkitt's Lymphoma Virus OR Burkitts Lymphoma Virus OR E-B Virus OR E B Virus OR E-B Viruses OR Infectious Mononucleosis Virus OR Infectious Mononucleosis Viruses OR Mononucleosis Virus, Infectious OR Mononucleosis Viruses, Infectious OR Epstein-Barr Virus OR Epstein Barr Virus OR Herpesvirus 4 (gamma), Human OR HHV-4 OR Human Herpesvirus 4 OR Burkitt Herpesvirus OR Herpesvirus, Burkitt OR EBV) AND ("Stomach Neoplasms"[Mesh] OR Neoplasm, Stomach OR Stomach Neoplasm OR Neoplasms, Stomach OR Gastric Neoplasms OR Gastric Neoplasm OR Neoplasm, Gastric OR Neoplasms, Gastric OR Cancer of Stomach OR Stomach Cancers OR Gastric Cancer OR Cancer, Gastric OR Cancers, Gastric OR Gastric Cancers OR Stomach Cancer OR Cancer, Stomach OR Cancers, Stomach OR Cancer of the Stomach) AND ("Prevalence"[Mesh] OR "Cross-Sectional Studies"[Mesh] OR "Epidemiology"[Mesh] OR "Case-Control Studies"[Mesh])

**Scopus**

TITLE-ABS-KEY ( "Herpesvirus 4, Human" OR "Burkitt Lymphoma Virus" OR "Lymphoma Virus, Burkitt" OR "Burkitt's Lymphoma Virus" OR "Burkitts Lymphoma Virus" OR "E-B Virus" OR "E B Virus" OR "E-B Viruses" OR "Infectious Mononucleosis Virus" OR "Infectious Mononucleosis Viruses" OR "Mononucleosis Virus, Infectious" OR "Mononucleosis Viruses, Infectious" OR "Epstein-Barr Virus" OR "Epstein Barr Virus" OR "Herpesvirus 4 (gamma), Human" OR "HHV-4" OR "Human Herpesvirus 4" OR "Burkitt Herpesvirus" OR "Herpesvirus, Burkitt" OR ebv ) AND ( "Stomach Neoplasms" OR "Neoplasm, Stomach" OR "Stomach Neoplasm" OR "Neoplasms, Stomach" OR "Gastric Neoplasms" OR "Gastric Neoplasm" OR "Neoplasm, Gastric" OR "Neoplasms, Gastric" OR "Cancer of Stomach" OR "Stomach Cancers" OR "Gastric Cancer" OR "Cancer, Gastric" OR "Cancers, Gastric" OR "Gastric Cancers" OR "Stomach Cancer" OR "Cancer, Stomach" OR "Cancers, Stomach" OR "Cancer of the Stomach" ) AND ( prevalence OR frequency OR "Cross-Sectional Studies" OR epidemiology OR "Case-Control Studies" ) AND ( LIMIT-TO ( DOCTYPE , "ar" ) ) AND ( LIMIT-TO ( SUBJAREA , "IMMU" ) )

**Web of sciences**

(TS=(("Herpesvirus 4, Human" OR "Burkitt Lymphoma Virus" OR "Lymphoma Virus, Burkitt" OR "Burkitt's Lymphoma Virus" OR "Burkitts Lymphoma Virus" OR "E-B Virus" OR "E B Virus" OR "E-B Viruses" OR "Infectious Mononucleosis Virus" OR "Infectious Mononucleosis Viruses" OR "Mononucleosis Virus, Infectious" OR "Mononucleosis Viruses, Infectious" OR "Epstein-Barr Virus" OR "Epstein Barr Virus" OR "Herpesvirus 4 (gamma), Human" OR "HHV-4" OR "Human Herpesvirus 4" OR "Burkitt Herpesvirus" OR "Herpesvirus, Burkitt" OR ebv ) AND ( "Stomach Neoplasms" OR "Neoplasm, Stomach" OR "Stomach Neoplasm" OR "Neoplasms, Stomach" OR "Gastric Neoplasms" OR "Gastric Neoplasm" OR "Neoplasm, Gastric" OR "Neoplasms, Gastric" OR "Cancer of Stomach" OR "Stomach Cancers" OR "Gastric Cancer" OR "Cancer, Gastric" OR "Cancers, Gastric" OR "Gastric Cancers" OR "Stomach Cancer" OR "Cancer, Stomach" OR "Cancers, Stomach" OR "Cancer of the Stomach" ) AND ("prevalence" OR "frequency" OR "Cross-Sectional Studies" OR "epidemiology" OR "Case-Control Studies"))) AND LANGUAGE: (English) AND DOCUMENT TYPES: (Article)
